# A novel nonlinear grey Bernoulli model NGBM(1,1,t^p,α) and its application in forecasting the express delivery volume per capita in China

**DOI:** 10.1371/journal.pone.0285460

**Published:** 2023-05-18

**Authors:** Maolin Cheng, Bin Liu

**Affiliations:** 1 Department of Statistics, Suzhou University of Science and Technology, Suzhou, PR China; 2 Department of Finance, Suzhou University of Science and Technology, Suzhou, PR China; Zhejiang Gongshang University, CHINA

## Abstract

The grey prediction is a common method in the prediction. Studies show that general grey models have high modeling precision when the time sequence varies slowly, but some grey models show low modeling precision for the high-growth sequence. The paper researches the grey modeling for the high-growth sequence using the extended nonlinear grey Bernoulli model NGBM(1,1,t⌃p,*α*). To improve the nonlinear grey Bernoulli model NGBM(1,1,t⌃p,*α*)’s prediction precision and make data have better adaptability to the model, the paper makes improvements in the following three aspects: (1) the paper improves the accumulated generating sequence of original time sequence, i.e. making a new transformation of traditional accumulated generating sequence; (2) the paper improves the model’s structure, extends the grey action and builds an extended nonlinear grey Bernoulli model NGBM(1,1,t⌃p,*α*); (3) the paper improves the model’s background value and uses the value of cubic spline function to approximate the background value. Because the parameters of the new accumulated generating sequence transformed, the nonlinear grey Bernoulli model’s time response equation and the background value are optimized simultaneously, the prediction precision increases greatly. The paper builds an extended nonlinear grey Bernoulli model NGBM(1,1,t⌃2,*α*) using the method proposed and seven comparison models for China’s express delivery volume per capita. Comparison results show that the extended nonlinear grey Bernoulli model built with the method proposed has high simulation and prediction precision and shows the precision superior to that of seven comparison models.

## Introduction

The grey system theory proposed by Chinese scholar Jvlong Deng in 1982 is mainly applied to the studies on problems with less data and poor information. The grey prediction is an important component of grey system theory. Currently, it has been used widely in economic fields like industry, agriculture and commerce, and other fields, such as environment, energy, society and military, but it has big prediction errors sometimes. In this case, to improve the grey model’s prediction precision, many scholars have made various studies on the grey prediction model. Cai et al. [1] proposed an asphalt pavement performance prediction method based on fractional grey model, which adopted fractional cumulative generation operation instead of traditional cumulative generation operation. Hu [2] proposed a GM(1,1) model based on residual correction for energy prediction. Different from the usual practice, the residual was not created independently. Experimental results showed that the proposed residual GM(1,1) model performed well compared with other residual GM(1,1) models. Cheng et al. [3] established a GM(1,1) model of China’s total industrial crude oil consumption by improving the parameter estimation of the model through four methods without changing the model structure. Liu et al. [4] proposed a constant stress accelerated degradation test and combined prediction model based on the Wiener process GM(1,1) model in order to predict the residual current device’s residual life. To improve the prediction accuracy of landslide deformation, Huang et al. [5] proposed an improved deformation prediction algorithm based on GM(1,1) model and empirical mode decomposition. To accurately predict medium—and long-term renewable energy generation, He [6] established a structurally adaptive discrete grey prediction model with new information priority. Wu et al. [7] studied CO2 emissions of BRICS countries (Brazil, Russia, India, China, and South Africa) by using a fractional non-homogeneous grey model, and the new model was developed to predict CO2 emissions of BRICS countries from 2019 to 2025. Wang et al. [8] proposed a new adaptive grey model of Caputo fractional derivative structure and a new Caputo fractional cumulative generator in order to accurately predict the future energy trend. By comparing optimization algorithms, particle swarm optimization algorithm was selected to optimize the model parameters. To predict photovoltaic energy, Yu et al. [9] developed a new grey system model, which takes into account the time-delay power effect with high flexibility, so as to deal more effectively with small and complex time series and has a more general formula. Three practical cases of energy prediction verified the effectiveness of the new model. The actual case showed that their proposed model was superior to the existing eight grey prediction models. Wang et al. [10] proposed a new adaptive grey Chebyshev polynomial Bernoulli model with fractional order structure to accurately predict renewable energy in China, which was based on NGBM(1,1) model to describe nonlinear phenomena. Xiang et al. [11] established the PM10 short-term prediction model using the grey system theory. They adopted Euler polynomial as the driver of the grey model, then adopted the convolution solution to make the new model computationally feasible, and adopted the grey wolf optimizer to optimize and select the nonlinear parameters of the model. Xiang et al. [12], aiming at the uncertainty of sewage discharge prediction in China, introduced the hyperbolic time delay term on the basis of grey system theory, established a new prediction model, determined the key nonlinear parameters of the model by grasshopper optimization algorithm, and proved the reliability of the model through a series of practical examples. The above scholars improved the grey model from different angles to improve the prediction accuracy.

The grey models generally used include the GM(1,1) model [13], the GM(1,N) model [14], the GM(N,1) model [15] and the nonlinear grey Bernoulli model [16, 17]. In the models, the nonlinear grey Bernoulli model is an important type, which has the strong adaptability to data fitting and thus has higher prediction precision generally. However, it’s hard to solve the power exponent and its parameters in the model, so the research on the nonlinear grey Bernoulli model is difficult and develops slowly.

Some scholars have researched the nonlinear grey Bernoulli model early. Wang et al. [18], starting from the whitening equation of the nonlinear grey Bernoulli model, solved the specific expression of the power index by using the grey derivative information covering principle, and discussed the relevant properties of the nonlinear grey Bernoulli model. However, the formula for solving the power index was relatively complex. Wang [19] improved the grey derivative of nonlinear grey Bernoulli model to improve the modeling accuracy. Wang et al. [20] optimized the power index and the parameters in the time-response formula of the nonlinear grey Bernoulli model through software programming to improve the modeling accuracy, but did not give the specific formula for solving the power index. Zhang and Chen [21] improved the modeling accuracy by processing the background value and initial conditions, but ignored the exponential part of the time response when they abstracted the curve into a non-homogeneous exponential function in the process of optimizing the background value. Hu [22] optimized the background value through data transformation. Compared with the traditional nonlinear grey Bernoulli model, although this method improved the modeling accuracy, it ignored the constant term in the time response.

In recent years, some scholars have made more in-depth theoretical and application studies on the nonlinear grey Bernoulli model. Cheng and Liu [23], Ma and Wei [24] extended the grey action amount of the traditional nonlinear grey Bernoulli model and proposed the extended nonlinear grey Bernoulli model, and gave the modeling method, and the examples showed that their proposed extended nonlinear grey Bernoulli model had high accuracy. Luo and Chen [25] proposed a nonlinear grey Bernoulli model with linear time-varying parameters for the prediction of non-equidistant time series. Zeng [26], Huang et al. [27] proposed a new nonlinear grey Bernoulli model building method to solve the problem that the traditional nonlinear grey Bernoulli model was not good at predicting the oscillation time series. Examples showed that their proposed model and method had high accuracy. Ding et al. [28] simultaneously optimized the power exponent and initial conditions of the nonlinear grey Bernoulli model, obtained the model parameter values by minimizing the average error, and verified the effectiveness and practicability of the model with an example. Zhou et al. [29] proposed a new time-varying grey Bernoulli model for accurate prediction of electric vehicles inventory and sales. They first designed time-varying parameters and power indices to explore the nonlinear development trend of the series, and then determined the optimal solution by using the cuckoo search algorithm. Wu et al. [30] proposed a new nonlinear grey Bernoulli model with a combinatorial component accumulation generator to predict oil consumption and end-consumption in China, considering the uncertainty and volatility of the petroleum system. The newly designed model combined the traditional fraction accumulation with the fitting fraction accumulation and introduced a combinatorial fraction accumulation generator. Compared with the old accumulation, the new optimized accumulation can improve the flexible mining ability of the development law of time series. Zeng et al. [31] proposed a matrix nonlinear grey Bernoulli model based on interval sequence in order to accurately predict power generation. To make the model directly applicable to interval sequence, the traditional grey Bernoulli model was linearized by power function transformation, then the parameters were matrixed, and finally the recursive prediction formula was obtained by Cramer’s law. The new model can be used to predict exponential interval series, saturation interval series and parabolic interval series. Yin and Mao [32] proposed a new fractional multivariate grey Bernoulli model for short-term power load prediction. The fractional differential equation and fractional cumulative generation sequence were integrated into the new model. Secondly, they improved the grey wolf algorithm, so that many parameters in the model were better optimized. Tong et al. [33], To accurately predict natural gas consumption, started from the traditional nonlinear grey Bernoulli model, expanded the development coefficient and grey action, proposed a new adaptive time-varying grey Bernoulli prediction model, derived the model’s time-response formula, and discussed the relationship between model parameters and model accuracy. Then, the nonlinear parameters and power parameters of the new model were optimized by genetic algorithm, and the validity of the model was analyzed by predicting the consumption of natural gas in China, the United States and Russia.

The scholars mainly improved the model in terms of grey derivative, power exponent, initial value, background value, model extension and parameter optimization to further improve the precision of model, but there were some defects in the modeling for some data, such as the high-growth sequence, manifested as the poor modeling precision. There are, essentially, the following three main factors affecting the modeling precision: first, the data should adapt to the variation law of model; second, the model should meet the variation characteristics of data; third, the method should be chosen properly. To improve the modeling precision for high-growth sequence, the paper makes improvements in the following three aspects: (1) the paper improves the accumulated generating sequence of original time sequence, i.e. making a new transformation of traditional accumulated generating sequence, to enhance the high-growth sequence’s adaptability to the model; (2) the paper improves the model’s structure and extends the grey action, i.e. building an extended nonlinear grey Bernoulli model NGBM(1,1,t⌃p,*α*), to make the model adapt to the variation of high-growth sequence; (3) the paper improves the model’s background value and uses the cubic spline function value to approximate the background value to enhance the estimation precision of parameter.

Because the parameters improved in the three aspects are optimized simultaneously, the precision of model increases greatly. To avoid big average simulation relative error (*MAPE*_1_) or big average prediction relative error (*MAPE*_2_) of the model, we make the objective function be the minimum of max(*MAPE*_1_, *MAPE*_2_).

It should be noticed that the improvement in the accumulated generating sequence of original time sequence has many transformation forms and the extension of the model’s grey action also has many manifestation forms, so how to find the proper form should be studied further.

The paper first gives the modeling method of traditional nonlinear grey Bernoulli model, then proposes the form of extended nonlinear grey Bernoulli model NGBM(1,1,t⌃p,*α*), next gives the method to build an extended nonlinear grey Bernoulli model NGBM(1,1,t⌃p,*α*), i.e. giving the time response equation for prediction and its parameter optimization method, and finally builds an extended nonlinear grey Bernoulli model NGBM(1,1,t⌃p,*α*) using the method proposed and other seven models for China’s express delivery volume per capita. Results show that the extended nonlinear grey Bernoulli model built with the method proposed has high simulation and prediction precision and shows the precision superior to that of other seven models in comparisons.

## Methods

### The method to build the traditional nonlinear grey Bernoulli model

Suppose the original time sequence is *x*^(0)^ = {*x*^(0)^(1), *x*^(0)^(2), ⋯, *x*^(0)^(*n*)} and its first-order accumulated generating sequence (1 − *AGO*) is *x*^(1)^ = {*x*^(1)^(1), *x*^(1)^(2), ⋯, *x*^(1)^(*n*)} in which $x^{\left(1\right)}\left(k\right)=\sum_{i=1}^{k}x^{\left(0\right)}\left(i\right),k=1,2,\cdots ,n$. Suppose the sequence *x*^(1)^ after the accumulation meets the following dynamic differential equation:

$$\begin{array}{c} \frac{dx^{\left(1\right)}\left(t\right)}{dt}+ax^{\left(1\right)}\left(t\right)=b{\left(x^{\left(1\right)}\left(t\right)\right)}^{\alpha }. \end{array}$$ (1)

Make an integration operation from both sides of the equation in [*k* − 1, *k*], then get

$$\begin{array}{c} x^{\left(1\right)}\left(k\right)-x^{\left(1\right)}\left(k-1\right)+a\int_{k-1}^{k}x^{\left(1\right)}\left(t\right)dt=b\int_{k-1}^{k}{\left(x^{\left(1\right)}\left(t\right)\right)}^{\alpha }dt, \end{array}$$ (2)

i.e. $x^{\left(0\right)}\left(k\right)+a\int_{k-1}^{k}x^{\left(1\right)}\left(t\right)dt=b\int_{k-1}^{k}{\left(x^{\left(1\right)}\left(t\right)\right)}^{\alpha }dt$.

Let $z^{\left(1\right)}\left(k\right)=\int_{k-1}^{k}x^{\left(1\right)}\left(t\right)dt$ and $z^{\left(2\right)}\left(k\right)=\int_{k-1}^{k}{\left(x^{\left(1\right)}\left(t\right)\right)}^{\alpha }dt$, and then have

$$\begin{array}{c} x^{\left(0\right)}\left(k\right)+az^{\left(1\right)}\left(k\right)=bz^{\left(2\right)}\left(k\right). \end{array}$$ (3)

The traditional nonlinear grey Bernoulli model writes *z*^(2)^(*k*) as (*z*^(1)^(*k*))^*α*^ approximately, then have

$$\begin{array}{c} x^{\left(0\right)}\left(k\right)+az^{\left(1\right)}\left(k\right)=b{\left(z^{\left(1\right)}\left(k\right)\right)}^{\alpha }. \end{array}$$ (4)

**Definition 1** [34]: Call *z*^(1)^ = {*z*^(1)^(1), *z*^(1)^(2), ⋯, *z*^(1)^(*n*)} the background value sequence of *x*^(1)^(*k*) in which $z^{\left(1\right)}\left(k\right)=\int_{k-1}^{k}x^{\left(1\right)}\left(t\right)dt=\alpha x^{\left(1\right)}\left(k-1\right)+\left(1-\alpha \right)x^{\left(1\right)}\left(k\right)$. Especially when $\alpha =\frac{1}{2}$, $z^{\left(1\right)}\left(k\right)=\frac{1}{2}\left(x^{\left(1\right)}\left(k-1\right)+x^{\left(1\right)}\left(k\right)\right)$.

**Definition 2** [34]: Call *x*^(0)^(*k*) + *az*^(1)^(*k*) = *b*(*z*^(1)^(*k*))^*α*^ the grey differential equation of nonlinear grey Bernoulli model.

**Definition 3** [34]: Call $\frac{dx^{\left(1\right)}\left(t\right)}{dt}+ax^{\left(1\right)}\left(t\right)=b{\left(x^{\left(1\right)}\left(t\right)\right)}^{\alpha }$ the whitening equation of nonlinear grey Bernoulli model.

**Theorem 1** [34]: The parameter estimates of nonlinear grey Bernoulli model are

$$\begin{array}{c} {}[\begin{array}{c} a\\ b \end{array}]={\left(B^{\prime }B\right)}^{-1}B^{\prime }Y, \end{array}$$ (5)

where

$$\begin{array}{c} Y=[\begin{array}{c} x^{\left(0\right)}\left(2\right)\\ x^{\left(0\right)}\left(3\right)\\ \vdots \\ x^{\left(0\right)}\left(n\right) \end{array}],B=[\begin{array}{cc} -z^{\left(1\right)}\left(2\right) & {\left(z^{\left(1\right)}\left(2\right)\right)}^{\alpha }\\ -z^{\left(1\right)}\left(3\right) & {\left(z^{\left(1\right)}\left(3\right)\right)}^{\alpha }\\ \vdots & \vdots \\ -z^{\left(1\right)}\left(n\right) & {\left(z^{\left(1\right)}\left(n\right)\right)}^{\alpha } \end{array}]. \end{array}$$ (6)

**Theorem 2** [34]: The time response function of nonlinear grey Bernoulli model is

$$\begin{array}{c} {\hat{x}}^{\left(1\right)}\left(t\right)=\{\frac{b}{a}+\left[x^{\left(1\right)}{\left(1\right)}^{\left(1-\alpha \right)}-\frac{b}{a}\right]e^{a(\alpha -1)(t-1)}{\}}^{\frac{1}{\alpha -1}}. \end{array}$$ (7)

The estimate of power exponent *α* can be obtained with the optimization method generally. According to the time response function sequence, we can get the simulation value and prediction value of original sequence from ${\hat{x}}^{\left(0\right)}\left(k\right)={\hat{x}}^{\left(1\right)}\left(k\right)-{\hat{x}}^{\left(1\right)}\left(k-1\right)$.

### The form of extended nonlinear grey Bernoulli model NGBM(1,1,t⌃p,*α*)

To make data have better adaptability to the model, the paper first improves the accumulated generating sequence of *x*^(0)^(*t*). Then, have the following definition.

**Definition 4** [35]: Suppose the original time sequence is *x*^(0)^ = {*x*^(0)^(1), *x*^(0)^(2), ⋯, *x*^(0)^(*n*)} and the new accumulated generating sequence is defined as *x*^(*r*)^ = {*x*^(*r*)^(1), *x*^(*r*)^(2), ⋯, *x*^(*r*)^(*n*)} in which $x^{\left(r\right)}\left(k\right)=\sum_{i=1}^{k}\frac{x^{\left(0\right)}\left(i\right)}{g[\text{sin}\left(\frac{\pi }{2}\left(1+he^{-vt}\right)\right]}$, *g* > 0, *h* ≥ 0, *v* ≥ 0, *k* = 1, 2, ⋯, *n*.

Especially when *g* = 1, *h* = 0, sequence *x*^(*r*)^ becomes *x*^(0)^, so for a specific grey model, if it meets the rules of traditional accumulated generating sequence, it must meet the rules of new accumulated generating sequence. Therefore, improving the accumulated generating sequence of *x*^(0)^(*t*) can improve modeling precision. In particular, the improvement in modeling accuracy is greater for high-growth sequences.

Next, the paper extends the structure of traditional nonlinear grey Bernoulli model, extending the grey action (*x*^(*r*)^(*t*))^*α*^ to be (*b*_0_ + *b*_1_*t* + *b*_2_*t*^2^+ ⋯ + *b*_*p*_*t*^*p*^)(*x*^(*r*)^(*t*))^*α*^ to make the model have the generality to be adaptive to more variations of data. Then, have the following definitions. In particular, the adaptability of high-growth sequences to the model is enhanced.

**Definition 5**: Suppose the original time sequence is *x*^(0)^ = {*x*^(0)^(1), *x*^(0)^(2), ⋯, *x*^(0)^(*n*)}, the new accumulated generating sequence is *x*^(*r*)^ = {*x*^(*r*)^(1), *x*^(*r*)^(2), ⋯, *x*^(*r*)^(*n*)} and the new background value is $z^{\left(r\right)}\left(k\right)=\int_{k-1}^{k}x^{\left(r\right)}\left(t\right)dt$. Call *x*^(*r*)^(*k*) + *az*^(*r*)^(*k*) = (*b*_0_ + *b*_1_*t* + *b*_2_*t*^2^ + ⋯ + *b*_*p*_*t*^*p*^)(*z*^(*r*)^(*k*))^*α*^ the grey differential equation of extended grey model NGBM(1,1,t⌃p,*α*).

**Definition 6**: Call $\frac{dx^{\left(r\right)}\left(t\right)}{dt}+ax^{\left(r\right)}\left(t\right)=\left(b_{0}+b_{1}t+b_{2}t^{2}+\cdots +b_{p}t^{p}\right){\left(x^{\left(r\right)}\left(t\right)\right)}^{\alpha }$ the whitening equation of extended grey model NGBM(1,1,t⌃p,*α*). Especially, when *p* = 0, the equation above is the whitening equation of traditional nonlinear grey Bernoulli model.

**Definition 7**: When *p* = 1, call $\frac{dx^{\left(r\right)}\left(t\right)}{dt}+ax^{\left(r\right)}\left(t\right)=\left(b_{0}+b_{1}t\right){\left(x^{\left(r\right)}\left(t\right)\right)}^{\alpha }$ the whitening equation of extended nonlinear grey Bernoulli model NGBM(1,1,t,*α*).

**Definition 8**: When *p* = 2, call $\frac{dx^{\left(r\right)}\left(t\right)}{dt}+ax^{\left(r\right)}\left(t\right)=\left(b_{0}+b_{1}t+b_{2}t^{2}\right){\left(x^{\left(r\right)}\left(t\right)\right)}^{\alpha }$ the whitening equation of extended nonlinear grey Bernoulli model NGBM(1,1,t⌃2,*α*).

### The time response equation of extended nonlinear grey Bernoulli model NGBM(1,1,t⌃p,*α*)

**Theorem 3**: Suppose sequence *x*^(*r*)^ = {*x*^(*r*)^(1), *x*^(*r*)^(2), ⋯, *x*^(*r*)^(*n*)}, and the whitening equation of extended nonlinear grey Bernoulli model NGBM(1,1,t⌃2,*α*) is $\frac{dx^{\left(r\right)}\left(t\right)}{dt}+ax^{\left(r\right)}\left(t\right)=\left(b_{0}+b_{1}t+b_{2}t^{2}\right){\left(x^{\left(r\right)}\left(t\right)\right)}^{\alpha }$, and then its time response equation is

$$\begin{array}{c} {\hat{x}}^{\left(r\right)}\left(t\right)=\{e^{-a(1-\alpha )(t-1)}[{\left(x^{\left(r\right)}\left(1\right)\right)}^{\left(1-\alpha \right)}+\left(1-\alpha \right)e^{-a(1-\alpha )}g\left(t\right)]{\}}^{\frac{1}{1-\alpha }}, \end{array}$$ (8)

where

$$\begin{array}{c} \begin{array}{c} g\left(t\right)=\frac{b_{2}t^{2}e^{a(1-\alpha )t}}{a(1-\alpha )}-\frac{2b_{2}te^{a(1-\alpha )t}}{a^{2}{\left(1-\alpha \right)}^{2}}+\frac{b_{1}te^{a(1-\alpha )}}{a(1-\alpha )}+\frac{2b_{2}e^{a(1-\alpha )t}}{a^{3}{\left(1-\alpha \right)}^{3}}-\frac{b_{1}e^{a(1-\alpha )t}}{a^{2}{\left(1-\alpha \right)}^{2}}+\frac{b_{0}e^{a(1-\alpha )t}}{a(1-\alpha )}\\ -\frac{2b_{2}e^{a(1-\alpha )}}{a^{3}{\left(1-\alpha \right)}^{3}}+\frac{2b_{2}e^{a(1-\alpha )}}{a^{2}{\left(1-\alpha \right)}^{2}}-\frac{b_{2}e^{a(1-\alpha )}}{a(1-\alpha )}+\frac{b_{1}e^{a(1-\alpha )}}{a^{2}{\left(1-\alpha \right)}^{2}}-\frac{e^{a(1-\alpha )}}{a(1-\alpha )}-\frac{b_{0}e^{a(1-\alpha )}}{a(1-\alpha )}. \end{array} \end{array}$$ (9)

**Proof**:

$$\begin{array}{c} \frac{dx^{\left(r\right)}\left(t\right)}{dt}+ax^{\left(r\right)}\left(t\right)=\left(b_{0}+b_{1}t+b_{2}t^{2}\right){\left(x^{\left(r\right)}\left(t\right)\right)}^{\alpha }, \end{array}$$ (10)

let *y* = (*x*^(*r*)^(*t*))^1−*α*^, and then get

$$\begin{array}{c} \frac{dy}{dt}=-\left(1-\alpha \right)ay+\left(1-\alpha \right)\left(b_{0}+b_{1}t+b_{2}t^{2}\right). \end{array}$$ (11)

Its solution is

$$\begin{array}{c} y\left(t\right)=e^{\int_{1}^{t}-a(1-\alpha )d\delta }\left[y\left(1\right)+\int_{1}^{t}e^{\int_{1}^{\theta }a(1-\alpha )d\delta }\left(1-\alpha \right)\left(b_{0}+b_{1}\theta +b_{2}\theta^{2}\right)d\theta \right], \end{array}$$ (12)

i.e.

$$\begin{array}{c} {\hat{x}}^{\left(r\right)}\left(t\right)=\{e^{-a(1-\alpha )(t-1)}[{\left(x^{\left(r\right)}\left(1\right)\right)}^{\left(1-\alpha \right)}+\left(1-\alpha \right)e^{-a(1-\alpha )}g\left(t\right)]{\}}^{\frac{1}{1-\alpha }}, \end{array}$$ (13)

where

$$\begin{array}{c} \begin{array}{c} g\left(t\right)=\frac{b_{2}t^{2}e^{a(1-\alpha )t}}{a(1-\alpha )}-\frac{2b_{2}te^{a(1-\alpha )t}}{a^{2}{\left(1-\alpha \right)}^{2}}+\frac{b_{1}te^{a(1-\alpha )}}{a(1-\alpha )}+\frac{2b_{2}e^{a(1-\alpha )t}}{a^{3}{\left(1-\alpha \right)}^{3}}-\frac{b_{1}e^{a(1-\alpha )t}}{a^{2}{\left(1-\alpha \right)}^{2}}+\frac{b_{0}e^{a(1-\alpha )t}}{a(1-\alpha )}\\ -\frac{2b_{2}e^{a(1-\alpha )}}{a^{3}{\left(1-\alpha \right)}^{3}}+\frac{2b_{2}e^{a(1-\alpha )}}{a^{2}{\left(1-\alpha \right)}^{2}}-\frac{b_{2}e^{a(1-\alpha )}}{a(1-\alpha )}+\frac{b_{1}e^{a(1-\alpha )}}{a^{2}{\left(1-\alpha \right)}^{2}}-\frac{e^{a(1-\alpha )}}{a(1-\alpha )}-\frac{b_{0}e^{a(1-\alpha )}}{a(1-\alpha )}. \end{array} \end{array}$$ (14)

### The parameter optimization method of extended nonlinear grey Bernoulli model NGBM(1,1,t⌃2,*α*)

**Theorem 4**: The whitening equation of extended nonlinear grey Bernoulli model NGBM(1,1,t⌃2,*α*) is

$$\begin{array}{c} \frac{dx^{\left(r\right)}\left(t\right)}{dt}+ax^{\left(r\right)}\left(t\right)=\left(b_{0}+b_{1}t+b_{2}t^{2}\right){\left(x^{\left(r\right)}\left(t\right)\right)}^{\alpha }. \end{array}$$ (15)

For the given *α*, *w*_1_, *w*_2_, *w*_3_, *w*_4_, *g*, *h*, *v*, have the parameter estimates

$$\begin{array}{c} {}[\begin{array}{c} a\\ b_{0}\\ b_{1}\\ b_{2} \end{array}]={\left(EF\right)}^{-1}E^{\prime }F, \end{array}$$ (16)

where

$$\begin{array}{c} E=[\begin{array}{cccc} -z^{\left(1\right)}\left(2\right) & z^{\left(2\right)}\left(2\right) & z^{\left(3\right)}\left(2\right) & z^{\left(4\right)}\left(2\right)\\ -z^{\left(1\right)}\left(3\right) & z^{\left(2\right)}\left(3\right) & z^{\left(3\right)}\left(3\right) & z^{\left(4\right)}\left(3\right)\\ \vdots & \vdots & \vdots & \vdots \\ -z^{\left(1\right)}\left(n\right) & z^{\left(2\right)}\left(n\right) & z^{\left(3\right)}\left(n\right) & z^{\left(4\right)}\left(n\right) \end{array}], \end{array}$$ (17)

$$\begin{array}{c} F=[\begin{array}{c} x^{\left(r\right)}\left(2\right)-x^{\left(r\right)}\left(1\right)\\ x^{\left(0\right)}\left(3\right)-x^{\left(r\right)}\left(2\right)\\ \vdots \\ x^{\left(0\right)}\left(n\right)-x^{\left(r\right)}\left(n-1\right) \end{array}]. \end{array}$$ (18)

$$\begin{array}{c} z^{\left(1\right)}\left(k\right)=a_{1k}{\left[1-w_{1}\right]}^{3}+b_{1k}{\left[1-w_{1}\right]}^{2}+c_{1k}\left[1-w_{1}\right]+d_{1k},\left(0\leq w_{1}\leq 1\right), \end{array}$$ (19)

$$\begin{array}{c} z^{\left(2\right)}\left(k\right)=a_{2k}{\left[1-w_{2}\right]}^{3}+b_{2k}{\left[1-w_{2}\right]}^{2}+c_{2k}\left[1-w_{2}\right]+d_{2k},\left(0\leq w_{2}\leq 1\right), \end{array}$$ (20)

$$\begin{array}{c} z^{\left(3\right)}\left(k\right)=a_{3k}{\left[1-w_{3}\right]}^{3}+b_{3k}{\left[1-w_{3}\right]}^{2}+c_{3k}\left[1-w_{3}\right]+d_{3k},\left(0\leq w_{3}\leq 1\right), \end{array}$$ (21)

$$\begin{array}{c} z^{\left(4\right)}\left(k\right)=a_{4k}{\left[1-w_{4}\right]}^{3}+b_{4k}{\left[1-w_{4}\right]}^{2}+c_{4k}\left[1-w_{4}\right]+d_{4k},\left(0\leq w_{4}\leq 1\right), \end{array}$$ (22)

and (*a*_*ik*_, *b*_*ik*_, *c*_*ik*_, *d*_*ik*_), (*i* = 1, 2, 3, 4; *k* = 2, 3⋯, *n*) is the cubic spline interpolation function coefficient.

**Proof**: The whitening equation of extended nonlinear grey Bernoulli model NGBM(1,1,t⌃2,*α*) is

$$\begin{array}{c} \frac{dx^{\left(r\right)}\left(t\right)}{dt}+ax^{\left(r\right)}\left(t\right)=b_{0}{\left(x^{\left(r\right)}\left(t\right)\right)}^{\alpha }+b_{1}t{\left(x^{\left(r\right)}\left(t\right)\right)}^{\alpha }+b_{2}t^{2}{\left(x^{\left(r\right)}\left(t\right)\right)}^{\alpha }. \end{array}$$ (23)

Make an integration operation from both sides of the equation in [*k* − 1, *k*], then get

$$\begin{array}{c} \begin{array}{c} \int_{k-1}^{k}\frac{dx^{\left(r\right)}\left(t\right)}{dt}dt+a\int_{k-1}^{k}x^{\left(r\right)}\left(t\right)dt\\ =b_{0}\int_{k-1}^{k}{\left(x^{\left(r\right)}\left(t\right)\right)}^{\alpha }dt+b_{1}\int_{k-1}^{k}t{\left(x^{\left(r\right)}\left(t\right)\right)}^{\alpha }dt+b_{2}\int_{k-1}^{k}t^{2}{\left(x^{\left(r\right)}\left(t\right)\right)}^{\alpha }dt, \end{array} \end{array}$$ (24)

let $z^{\left(1\right)}\left(k\right)=\int_{k-1}^{k}x^{\left(r\right)}\left(t\right)dt$, $z^{\left(2\right)}\left(k\right)=\int_{k-1}^{k}(x^{\left(r\right)}\left(t\right){)}^{\alpha }dt$, $z^{\left(3\right)}\left(k\right)=\int_{k-1}^{k}t(x^{\left(r\right)}\left(t\right){)}^{\alpha }dt$, $z^{\left(4\right)}\left(k\right)=\int_{k-1}^{k}t^{2}(x^{\left(r\right)}\left(t\right){)}^{\alpha }dt$.

Apparently,

$$\begin{array}{c} z^{\left(1\right)}\left(k\right)=\int_{k-1}^{k}x^{\left(r\right)}\left(t\right)dt=x^{\left(r\right)}\left(\xi \right),\left(k-1\leq \xi \leq k\right), \end{array}$$ (25)

$$\begin{array}{c} z^{\left(2\right)}\left(k\right)=\int_{k-1}^{k}(x^{\left(r\right)}\left(t\right){)}^{\alpha }dt={\left(x^{\left(r\right)}\left(\eta \right)\right)}^{\alpha },\left(k-1\leq \eta \leq k\right), \end{array}$$ (26)

$$\begin{array}{c} z^{\left(3\right)}\left(k\right)=\int_{k-1}^{k}t(x^{\left(r\right)}\left(t\right){)}^{\alpha }dt=\varsigma {\left(x^{\left(r\right)}\left(\varsigma \right)\right)}^{\alpha },\left(k-1\leq \varsigma \leq k\right), \end{array}$$ (27)

$$\begin{array}{c} z^{\left(4\right)}\left(k\right)=\int_{k-1}^{k}t^{2}(x^{\left(r\right)}\left(t\right){)}^{\alpha }dt=\psi^{2}{\left(x^{\left(r\right)}\left(\psi \right)\right)}^{\alpha },\left(k-1\leq \psi \leq k\right). \end{array}$$ (28)

In this case, the background value function is an interpolation function, and choose the cubic spline interpolation function as the interpolation function.

If the background value function is

$$\begin{array}{c} x^{\left(r\right)}\left(t\right)=\{x_{k}^{\left(r\right)}\left(t\right),t\in \left[k-1,k\right],k=2,3,\cdots ,N-1\}, \end{array}$$ (29)

where $x_{k}^{\left(r\right)}\left(t\right)=a_{1k}{\left[t-\left(k-1\right)\right]}^{3}+b_{1k}{\left[t-\left(k-1\right)\right]}^{2}+c_{1k}\left[t-\left(k-1\right)\right]+d_{1k}$, and (*a*_1*k*_, *b*_1*k*_, *c*_1*k*_, *d*_1*k*_) is a known cubic spline interpolation function coefficient.

Apparently, the spline interpolation function meets when *t* = *k* − 1, *x*^(*r*)^(*t*) = *x*^(*r*)^(*k* − 1); when *t* = *k*, *x*^(*r*)^(*t*) = *x*^(*r*)^(*k*).

Then, the background value is as follows:

$$\begin{array}{c} \begin{array}{c} z^{\left(1\right)}\left(k\right)=x^{\left(r\right)}\left(\xi \right)=a_{1k}{\left[\xi -\left(k-1\right)\right]}^{3}+b_{1k}{\left[\xi -\left(k-1\right)\right]}^{2}+c_{1k}\left[\xi -\left(k-1\right)\right]+d_{1k}\\ =a_{1k}{\left[k-w_{1}-\left(k-1\right)\right]}^{3}+b_{1k}{\left[k-w_{1}-\left(k-1\right)\right]}^{2}+c_{1k}\left[k-w_{1}-\left(k-1\right)\right]+d_{1k}\\ =a_{1k}{\left[1-w_{1}\right]}^{3}+b_{1k}{\left[1-w_{1}\right]}^{2}+c_{1k}\left[1-w_{1}\right]+d_{1k}, \end{array} \end{array}$$ (30)

$$\begin{array}{c} (0\leq w_{1}\leq 1). \end{array}$$ (31)

Similarly, if the background value function is

$$\begin{array}{c} {\left(x^{\left(r\right)}\left(t\right)\right)}^{\alpha }=\{{\left(x_{k}^{\left(r\right)}\left(t\right)\right)}^{\alpha },t\in \left[k-1,k\right],k=2,3,\cdots ,N-1\}, \end{array}$$ (32)

where ${\left(x_{k}^{\left(r\right)}\left(t\right)\right)}^{\alpha }=a_{2k}{\left[t-\left(k-1\right)\right]}^{3}+b_{2k}{\left[t-\left(k-1\right)\right]}^{2}+c_{2k}\left[t-\left(k-1\right)\right]+d_{2k}$, and (*a*_2*k*_, *b*_2*k*_, *c*_2*k*_, *d*_2*k*_) is the known cubic spline interpolation function coefficient, the background value is as follows:

$$\begin{array}{c} \begin{array}{c} z^{\left(2\right)}\left(k\right)={\left(x^{\left(r\right)}\left(\eta \right)\right)}^{\alpha }\\ =a_{2k}{\left[1-w_{2}\right]}^{3}+b_{2k}{\left[1-w_{2}\right]}^{2}+c_{2k}\left[1-w_{2}\right]+d_{2k}, \end{array} \end{array}$$ (33)

$$\begin{array}{c} (0\leq w_{2}\leq 1). \end{array}$$ (34)

If the background value function is

$$\begin{array}{c} t{\left(x^{\left(r\right)}\left(t\right)\right)}^{\alpha }=\{t{\left(x_{k}^{\left(r\right)}\left(t\right)\right)}^{\alpha },t\in \left[k-1,k\right],k=2,3,\cdots ,N-1\}, \end{array}$$ (35)

where $t{\left(x_{k}^{\left(r\right)}\left(t\right)\right)}^{\alpha }=a_{3k}{\left[t-\left(k-1\right)\right]}^{3}+b_{3k}{\left[t-\left(k-1\right)\right]}^{2}+c_{3k}\left[t-\left(k-1\right)\right]+d_{3k}$ and (*a*_3*k*_, *b*_3*k*_, *c*_3*k*_, *d*_3*k*_) is the known cubic spline interpolation function coefficient, the background value is as follows

$$\begin{array}{c} \begin{array}{c} z^{\left(3\right)}\left(k\right)=\varsigma {\left(x^{\left(r\right)}\left(\varsigma \right)\right)}^{\alpha },\\ =a_{3k}{\left[1-w_{3}\right]}^{3}+b_{3k}{\left[1-w_{3}\right]}^{2}+c_{3k}\left[1-w_{3}\right]+d_{3k}, \end{array} \end{array}$$ (36)

$$\begin{array}{c} (0\leq w_{3}\leq 1). \end{array}$$ (37)

If the background value function is

$$\begin{array}{c} t^{2}{\left(x^{\left(r\right)}\left(t\right)\right)}^{\alpha }=\{t^{2}{\left(x_{k}^{\left(r\right)}\left(t\right)\right)}^{\alpha },t\in \left[k-1,k\right],k=2,3,\cdots ,N-1\}, \end{array}$$ (38)

where $t^{2}{\left(x_{k}^{\left(r\right)}\left(t\right)\right)}^{\alpha }=a_{4k}{\left[t-\left(k-1\right)\right]}^{3}+b_{4k}{\left[t-\left(k-1\right)\right]}^{2}+c_{4k}\left[t-\left(k-1\right)\right]+d_{4k}$, and (*a*_4*k*_, *b*_4*k*_, *c*_4*k*_, *d*_4*k*_) is the known cubic spline interpolation function coefficient, the background value is as follows

$$\begin{array}{c} \begin{array}{c} z^{\left(4\right)}\left(k\right)=\psi^{2}{\left(x^{\left(r\right)}\left(\psi \right)\right)}^{\alpha }\\ =a_{4k}{\left[1-w_{4}\right]}^{3}+b_{4k}{\left[1-w_{4}\right]}^{2}+c_{4k}\left[1-w_{4}\right]+d_{4k}, \end{array} \end{array}$$ (39)

$$\begin{array}{c} (0\leq w_{4}\leq 1), \end{array}$$ (40)

and then have

$$\begin{array}{c} x^{\left(r\right)}\left(k\right)-x^{\left(r\right)}\left(k-1\right)=-az^{\left(1\right)}\left(k\right)+b_{0}z^{\left(2\right)}\left(k\right)+b_{1}z^{\left(3\right)}\left(k\right)+b_{2}z^{\left(4\right)}\left(k\right)+\varepsilon \left(k\right), \end{array}$$ (41)

where *ε*(*k*) is the error terms.

For the given *α*, *w*_1_, *w*_2_, *w*_3_, *w*_4_, *g*, *h*, *v*, get the parameter estimates (*a*, *b*_0_, *b*_1_, *b*_2_) with the least squares method:

$$\begin{array}{c} {}[\begin{array}{c} a\\ b_{0}\\ b_{1}\\ b_{2} \end{array}]={\left(EF\right)}^{-1}E^{\prime }F, \end{array}$$ (42)

where

$$\begin{array}{c} E=[\begin{array}{cccc} -z^{\left(1\right)}\left(2\right) & z^{\left(2\right)}\left(2\right) & z^{\left(3\right)}\left(2\right) & z^{\left(4\right)}\left(2\right)\\ -z^{\left(1\right)}\left(3\right) & z^{\left(2\right)}\left(3\right) & z^{\left(3\right)}\left(3\right) & z^{\left(4\right)}\left(3\right)\\ \vdots & \vdots & \vdots & \vdots \\ -z^{\left(1\right)}\left(n\right) & z^{\left(2\right)}\left(n\right) & z^{\left(3\right)}\left(n\right) & z^{\left(4\right)}\left(n\right) \end{array}], \end{array}$$ (43)

$$\begin{array}{c} F=[\begin{array}{c} x^{\left(r\right)}\left(2\right)-x^{\left(r\right)}\left(1\right)\\ x^{\left(0\right)}\left(3\right)-x^{\left(r\right)}\left(2\right)\\ \vdots \\ x^{\left(0\right)}\left(n\right)-x^{\left(r\right)}\left(n-1\right) \end{array}]. \end{array}$$ (44)

The formula for finding the simulated and predicted values of *x*^(0)^ is given below.

Because $x^{\left(r\right)}\left(k\right)=\sum_{i=1}^{k}\frac{x^{\left(0\right)}\left(i\right)}{g[\text{sin}\left(\frac{\pi }{2}\left(1+he^{-vi}\right)\right]},k=1,2,\cdots ,n$,

$$\begin{array}{c} \begin{array}{c} {\hat{x}}^{\left(r\right)}\left(k\right)-{\hat{x}}^{\left(r\right)}\left(k-1\right)=\sum_{i=1}^{k}\frac{x^{\left(0\right)}\left(i\right)}{g[\text{sin}\left(\frac{\pi }{2}\left(1+he^{-vi}\right)\right]}-\sum_{i=1}^{k-1}\frac{x^{\left(0\right)}\left(i\right)}{g[\text{sin}\left(\frac{\pi }{2}\left(1+he^{-vi}\right)\right]}\\ =\frac{x^{\left(0\right)}\left(k\right)}{g[\text{sin}\left(\frac{\pi }{2}\left(1+he^{-vk}\right)\right]}, \end{array} \end{array}$$ (45)

So *x*^(0)^’s simulation value is

$$\begin{array}{c} {\hat{x}}^{\left(0\right)}\left(k\right)=\left({\hat{x}}^{\left(r\right)}\left(k\right)-{\hat{x}}^{\left(r\right)}\left(k-1\right)\right)\cdot g[\text{sin}\left(\frac{\pi }{2}\left(1+he^{-vt}\right)\right],\left(k=2,3,\cdots ,n\right), \end{array}$$ (46)

and prediction value is

$$\begin{array}{c} {\hat{x}}^{\left(0\right)}\left(k\right)=\left({\hat{x}}^{\left(r\right)}\left(k\right)-{\hat{x}}^{\left(r\right)}\left(k-1\right)\right)\cdot g[\text{sin}\left(\frac{\pi }{2}\left(1+he^{-vt}\right)\right],\left(k=n+1,\cdots ,N\right). \end{array}$$ (47)

If all parameters are known, we can make simulations and predictions with the model. Suppose there are observation data of *N* years in which the data from year 1 to year *n* are used for modeling and the data from year *n* + 1 to year *N* are used for prediction. For *x*^(0)^, the simulation value and prediction value are recorded as ${\hat{x}}^{\left(0\right)}\left(k\right),\left(k=2,3,\cdots ,n\right)$ and ${\hat{x}}^{\left(0\right)}\left(k\right),\left(k=n+1,n+2,\cdots ,N\right)$ respectively; the average simulation relative error is $MAPE_{1}=\frac{1}{n-1}\sum_{k=2}^{n}|\frac{x^{\left(0\right)}\left(k\right)-{\hat{x}}^{\left(0\right)}\left(k\right)}{x^{\left(0\right)}\left(k\right)}|\times 100\%$; the average prediction relative error is $MAPE_{2}=\frac{1}{N-n}\sum_{k=n+1}^{N}|\frac{x^{\left(0\right)}\left(k\right)-{\hat{x}}^{\left(0\right)}\left(k\right)}{x^{\left(0\right)}\left(k\right)}|\times 100\%$.

In fact, the values of *α*, *w*_1_, *w*_2_, *w*_3_, *w*_4_, *g*, *h*, *v* are required, which can be determined using an optimization method, i.e. the following optimization problem:

$$\begin{array}{c} \underset{\alpha ,w_{1},w_{2},w_{3},w_{4},g,h,v}{\text{min}}\left(\text{max}\left(MAPE_{1},MAPE_{2}\right)\right), \end{array}$$ (48)

$$\begin{array}{c} s.t.\{\begin{array}{c} MAPE_{1}=\frac{1}{n-1}\sum_{k=2}^{n}|\frac{x^{\left(0\right)}\left(k\right)-{\hat{x}}^{\left(0\right)}\left(k\right)}{x^{\left(0\right)}\left(k\right)}|\times 100\%,\\ MAPE_{2}=\frac{1}{N-n}\sum_{k=n+1}^{N}|\frac{x^{\left(0\right)}\left(k\right)-{\hat{x}}^{\left(0\right)}\left(k\right)}{x^{\left(0\right)}\left(k\right)}|\times 100\%,\\ {\hat{x}}^{\left(0\right)}\left(t\right)=\left({\hat{x}}^{\left(r\right)}\left(t\right)-{\hat{x}}^{\left(r\right)}\left(t-1\right)\right)g[\text{sin}\left(\frac{\pi }{2}\left(1+he^{-vt}\right)\right],\\ {\hat{x}}^{\left(r\right)}\left(t\right)=\{e^{-a(1-\alpha )(t-1)}[{\left(x^{\left(r\right)}\left(1\right)\right)}^{\left(1-\alpha \right)}+\left(1-\alpha \right)e^{-a(1-\alpha )}g\left(t\right)]{\}}^{\frac{1}{1-\alpha }},\\ g\left(t\right)=\frac{b_{2}t^{2}e^{a(1-\alpha )t}}{a(1-\alpha )}-\frac{2b_{2}te^{a(1-\alpha )t}}{a^{2}{\left(1-\alpha \right)}^{2}}+\frac{b_{1}te^{a(1-\alpha )}}{a(1-\alpha )}+\frac{2b_{2}e^{a(1-\alpha )t}}{a^{3}{\left(1-\alpha \right)}^{3}}-\frac{b_{1}e^{a(1-\alpha )t}}{a^{2}{\left(1-\alpha \right)}^{2}}+\frac{b_{0}e^{a(1-\alpha )t}}{a(1-\alpha )}\\ -\frac{2b_{2}e^{a(1-\alpha )}}{a^{3}{\left(1-\alpha \right)}^{3}}+\frac{2b_{2}e^{a(1-\alpha )}}{a^{2}{\left(1-\alpha \right)}^{2}}-\frac{b_{2}e^{a(1-\alpha )}}{a(1-\alpha )}+\frac{b_{1}e^{a(1-\alpha )}}{a^{2}{\left(1-\alpha \right)}^{2}}-\frac{e^{a(1-\alpha )}}{a(1-\alpha )}-\frac{b_{0}e^{a(1-\alpha )}}{a(1-\alpha )},\\ {\left(a,b_{0},b_{1},b_{2}\right)}^{\prime }={\left(E^{\prime }F\right)}^{-1}E^{\prime }F,\\ \alpha \neq 0,1;0\leq w_{1},w_{2},w_{3},w_{4},\leq 1;g>0,h\geq 0,v\geq 0. \end{array} \end{array}$$ (49)

This optimization can be solved by MATLAB software using the global optimization command. Estimate the parameters and then get the simulation values and prediction values of *x*^(0)^ from ${\hat{x}}^{\left(0\right)}\left(k\right)=\left({\hat{x}}^{\left(r\right)}\left(k\right)-{\hat{x}}^{\left(r\right)}\left(k-1\right)\right)\cdot g[\text{sin}\left(\frac{\pi }{2}\left(1+he^{-vt}\right)\right]$.

## Application of grey model in forecasting express delivery volume per capita in China

China’s express delivery industry has been growing rapidly since year 2007. In 2021, the business volume of China’s express delivery service enterprises has reached 0.1083 trillion increasing by 29.9% year on year; the business income has reached 1033.23 billion yuan increasing by 17.5% year on year. China’s express delivery business shows an increasing contribution to economic growth. With the growth of gross express delivery, the express delivery volume per capita is growing rapidly. The express delivery volume reflects people’s living consumption and social & economic development levels and its future trend is cared about by everyone, so predicting China’s express delivery volume per capita accurately has great significance. The paper builds a grey prediction model for China’s express delivery volume per capita using the extended nonlinear grey Bernoulli model NGBM(1,1,t⌃p,*α*) proposed. China’s express delivery volume per capita is written as *x*^(0)^(*t*). See Table 1 for the specific data.

**Table 1:** Calculation results of grey modeling for China’s express delivery volume per capita.

Year	No.	*x*^(0)^(*t*)	Traditional nonlinear grey Bernoulli model		NGBM(1,1,t⌃2,*α*) model proposed	
			Simulation value	Relative error %	Simulation value	Relative error %
2007	1	0.9096	-	-	-	-
2008	2	1.1395	0.6203	45.5660	1.1391	0.0402
2009	3	1.3922	1.0275	26.1951	1.3323	4.3004
2010	4	1.7443	1.6840	3.4572	1.7842	2.2886
2011	5	2.7225	2.7225	-0.0000	2.7219	0.0233
2012	6	4.1829	4.3262	-3.4271	4.2968	2.7221
2013	7	6.7191	6.7279	-0.1314	6.6628	0.8384
2014	8	10.1414	10.1884	-0.4629	9.9883	1.5093
2015	9	14.9403	14.9403	0.0000	14.4455	3.3118
2016	10	22.4684	21.0905	6.1324	20.1991	10.0999
2017	11	28.6091	28.4938	0.4030	27.4011	4.2225
2018	12	36.0823	36.6497	-1.5725	36.1903	0.2993
			Prediction value	Relative error %	Prediction value	Relative error %
2019	13	45.0492	44.6957	0.7845	46.6930	3.6489
2020	14	59.0303	51.5563	12.6613	59.0250	0.0090
2021	15	76.6671	56.2254	26.6629	73.2937	4.4001
Average simulation relative error			-	7.94	-	2.70
Average prediction relative error			-	13.31	-	2.69
Average total relative error			-	9.09	-	2.70

First, build a traditional nonlinear grey Bernoulli model for original sequence and then get the parameter estimates through calculation:

$$\begin{array}{c} (a,b,\alpha )=(-0.5401,-0.0185,1.5138). \end{array}$$ (50)

The time response equation is

$$\begin{array}{c} \begin{array}{c} {\hat{x}}^{\left(1\right)}\left(t\right)=\{e^{-a(1-\alpha )(t-1)}[{\left(x^{\left(1\right)}\left(1\right)\right)}^{\left(1-\alpha \right)}+\left(1-\alpha \right)e^{-a(1-\alpha )}\left(\frac{be^{a(1-\alpha )t}}{a(1-\alpha )}-\frac{be^{a(1-\alpha )}}{a(1-\alpha )}\right)]{\}}^{\frac{1}{1-\alpha }}\\ =\{e^{-0.2775(t-1)}[1.0499-0.3893(-0.0666e^{0.2775t}+0.0879)]{\}}^{-1.9462}. \end{array} \end{array}$$ (51)

Calculate and get the simulation values and prediction values of original sequence in these periods with ${\hat{x}}^{\left(0\right)}\left(t\right)={\hat{x}}^{\left(1\right)}\left(t\right)-{\hat{x}}^{\left(1\right)}\left(t-1\right)$; calculate and get the relative errors of simulation values and prediction values of original sequence in the periods with $RE\left(t\right)=|\frac{x^{\left(0\right)}\left(t\right)-{\hat{x}}^{\left(0\right)}\left(t\right)}{x^{\left(0\right)}\left(t\right)}|\times 100\%$; calculate and get the average simulation relative error with $MAPE_{1}=\frac{1}{n-1}\sum_{k=2}^{n}|\frac{x^{\left(0\right)}\left(k\right)-{\hat{x}}^{\left(0\right)}\left(k\right)}{x^{\left(0\right)}\left(k\right)}|\times 100\%$ and calculate and get the average prediction relative error with $MAPE_{2}=\frac{1}{N-n}\sum_{k=n+1}^{N}|\frac{x^{\left(0\right)}\left(k\right)-{\hat{x}}^{\left(0\right)}\left(k\right)}{x^{\left(0\right)}\left(k\right)}|\times 100\%$. See Table 1 for the results.

Next, build the extended nonlinear grey Bernoulli model NGBM(1,1,t⌃2,*α*) proposed, i.e. the following model

$$\begin{array}{c} \frac{dx^{\left(1\right)}\left(t\right)}{dt}+ax^{\left(1\right)}\left(t\right)=\left(b_{0}+b_{1}t+b_{2}t^{2}\right){\left(x^{\left(1\right)}\left(t\right)\right)}^{\alpha }. \end{array}$$ (52)

Calculate and get the following parameter estimates using the method proposed:

$$\begin{array}{c} \begin{array}{c} \left(\alpha ,w_{1},w_{2},w_{3},w_{4},g,h,v\right)\\ =(0.4225,0.1314,0.3723,0.2485,0.1766,0.1228,0.9387,0.2130), \end{array} \end{array}$$ (53)

$$\begin{array}{c} \left(a,b_{0},b_{1},b_{2}\right)=(0.6703,9.5284,-1.2766,0.4257). \end{array}$$ (54)

Then, get the following time response equation:

$$\begin{array}{c} \begin{array}{c} {\hat{x}}^{\left(1\right)}\left(t\right)=\{e^{-a(1-\alpha )(t-1)}[{\left(x^{\left(1\right)}\left(1\right)\right)}^{\left(1-\alpha \right)}+\left(1-\alpha \right)e^{-a(1-\alpha )}h\left(t\right)]{\}}^{\frac{1}{1-\alpha }}\\ =\{e^{-0.3871(t-1)}[5.6429+0.3921g(t)]{\}}^{1.7317}, \end{array} \end{array}$$ (55)

where

$$\begin{array}{c} \begin{array}{c} g\left(t\right)=\frac{b_{2}t^{2}e^{a(1-\alpha )t}}{a(1-\alpha )}-\frac{2b_{2}te^{a(1-\alpha )t}}{a^{2}{\left(1-\alpha \right)}^{2}}+\frac{b_{1}te^{a(1-\alpha )t}}{a(1-\alpha )}+\frac{2b_{2}e^{a(1-\alpha )t}}{a^{3}{\left(1-\alpha \right)}^{3}}-\frac{b_{1}e^{a(1-\alpha )t}}{a^{2}{\left(1-\alpha \right)}^{2}}+\frac{b_{0}e^{a(1-\alpha )t}}{a(1-\alpha )}\\ -\frac{2b_{2}e^{a(1-\alpha )}}{a^{3}{\left(1-\alpha \right)}^{3}}+\frac{2b_{2}e^{a(1-\alpha )}}{a^{2}{\left(1-\alpha \right)}^{2}}-\frac{b_{2}e^{a(1-\alpha )}}{a(1-\alpha )}+\frac{b_{1}e^{a(1-\alpha )}}{a^{2}{\left(1-\alpha \right)}^{2}}-\frac{e^{a(1-\alpha )}}{a(1-\alpha )}-\frac{b_{0}e^{a(1-\alpha )}}{a(1-\alpha )}\\ =1.0998t^{2}e^{0.3871t}-8.9808te^{0.3871t}+47.8184e^{0.3871t}-58.8143. \end{array} \end{array}$$ (56)

Calculate and get the simulation values and prediction values of original sequence in these periods with ${\hat{x}}^{\left(0\right)}\left(k\right)=\left({\hat{x}}^{\left(r\right)}\left(k\right)-{\hat{x}}^{\left(r\right)}\left(k-1\right)\right)\cdot g[\text{sin}\left(\frac{\pi }{2}\left(1+he^{-vk}\right)\right]$ and the results are shown in Table 1. See Table 1 for the relative errors and average relative errors in the periods.

To compare the method proposed with the grey models proposed in other referencing documents in terms of modeling precision, the paper makes calculations.

Build a model using the improved method of extended nonlinear grey Bernoulli model given by referencing document [23] and then get the following time response equation:

$$\begin{array}{c} {\hat{x}}^{\left(1\right)}\left(t\right)=\{e^{-0.6245(t-1)}[\begin{array}{c} 0.9467+0.3100(4.1057t^{2}e^{0.6245t}\\ -1.6270te^{0.6245t}+6.7111e^{0.6245t}-10.0360) \end{array}]{\}}^{1.7275}. \end{array}$$ (57)

Calculate and get the simulation value and prediction value of original sequence with ${\hat{x}}^{\left(0\right)}\left(k\right)={\hat{x}}^{\left(1\right)}\left(k\right)-{\hat{x}}^{\left(1\right)}\left(k-1\right)$, and the results are shown in Table 2. Table 2 shows the relative errors and average relative error in the periods.

**Table 2:** Calculation results of grey models for China’s express delivery volume per capita built with methods proposed by other referencing documents.

Year	No.	*x*^(0)^(*t*)	Model of referencing document [23]		Model of referencing document [36]	
			Simulation value	Relative error %	Simulation value	Relative error %
2007	1	0.9096	-	-	-	-
2008	2	1.1395	1.1395	0.0000	0.7367	35.3464
2009	3	1.3922	1.1276	19.0037	1.2972	6.8186
2010	4	1.7443	1.6134	7.5020	2.1210	21.5951
2011	5	2.7225	2.6804	1.5462	3.2930	20.9554
2012	6	4.1829	4.4320	5.9553	4.9222	17.6737
2013	7	6.7191	7.0093	4.3186	7.1466	6.3623
2014	8	10.1414	10.5568	4.0962	10.1414	0.0003
2015	9	14.9403	15.2066	1.7824	14.1282	5.4361
2016	10	22.4684	21.0754	6.1998	19.3859	13.7192
2017	11	28.6091	28.2666	1.1972	26.2658	8.1909
2018	12	36.0823	36.8739	2.1939	35.2086	2.4213
			Prediction value	Relative error %	Prediction value	Relative error %
2019	13	45.0492	46.9830	4.2928	46.7670	3.8132
2020	14	59.0303	58.6737	0.6041	61.6319	4.4073
2021	15	76.6671	72.0207	6.0605	80.6672	5.2174
Average simulation relative error			-	4.89	-	12.59
Average prediction relative error			-	4.88	-	4.48
Average total relative error			-	4.89	-	10.85

Build a model using the improved method of nonlinear grey Bernoulli model given by referencing document [36] and then get the following time response equation:

$$\begin{array}{c} {\hat{x}}^{\left(1\right)}\left(k\right)=0.6275(4.3450e^{0.0690(k-1)}-3{.3762)}^{2.9827}. \end{array}$$ (58)

Calculate and get the simulation value and prediction value of original sequence with ${\hat{x}}^{\left(0\right)}\left(t\right)={\hat{x}}^{\left(1\right)}\left(t\right)-{\hat{x}}^{\left(1\right)}\left(t-1\right)$, and the results are shown in Table 2. Table 2 shows the relative errors and average relative error in the periods.

We build a model for $x_{\;}^{\left(0\right)}$ using the improved method of simultaneous grey model given by referencing document [37] and then get the following time response equation through calculations:

$$\begin{array}{c} \begin{array}{c} {\hat{x}}^{\left(1\right)}\left(t\right)=-exp\left(t*\left(0.3023943-0.2138529i\right)\right)*(exp\left(t*\left(-0.3023943+0.2138529i\right)\right)\\ {}*\left(719.9555+1664.616i\right)+t^{2}*exp\left(t*\left(-0.3023943+0.2138529i\right)\right)\\ {}*(1241.324+1249.708i)+t*exp(t*(-0.3023943+0.2138529i))\\ {}*(7958.549+10101.71i)+435.6867-866.8401i)\\ {}*(0.00153006+0.001373668i)-exp(t*(0.3023943+0.2138529i))\\ {}*(exp(t*(-0.3023943-0.2138529i))*(719.9555-1664.616i)\\ +t^{2}*exp\left(t*\left(-0.3023943-0.2138529i\right)\right)*\left(1241.324-1249.708i\right)\\ +t*exp(t*(-0.3023943-0.2138529i))*(7958.549-10101.71i)\\ +435.6867+866.8401i)*(0.00153006-0.001373668i). \end{array} \end{array}$$ (59)

We calculate and get the simulation and prediction values of original sequence in these periods with ${\hat{x}}^{\left(0\right)}\left(t\right)={\hat{x}}^{\left(1\right)}\left(t\right)-{\hat{x}}^{\left(1\right)}\left(t-1\right)$, and the results are shown in Table 3. See Table 3 for the relative errors and average relative errors in the periods.

**Table 3:** Calculation results of grey models for China’s express delivery volume per capita built with the methods proposed by other referencing documents.

Year	No.	*x*^(0)^(*t*)	Model of referencing document [37]		Model of referencing document [38]	
			Simulation value	Relative error %	Simulation value	Relative error %
2007	1	0.9096	-	-	-	-
2008	2	1.1395	1.7138	50.3940	-	-
2009	3	1.3922	1.4408	3.4954	2.1414	53.8191
2010	4	1.7443	1.5726	9.8424	2.5851	48.2045
2011	5	2.7225	2.2846	16.0840	3.1774	16.7090
2012	6	4.1829	3.7903	9.3854	4.5133	7.8998
2013	7	6.7191	6.3324	5.7553	6.4682	3.7334
2014	8	10.1414	10.1622	0.2053	9.7824	3.5405
2015	9	14.9403	15.5001	3.7468	14.2121	4.8742
2016	10	22.4684	22.4684	0.0000	20.4142	9.1427
2017	11	28.6091	30.9871	8.3121	30.1367	5.3395
2018	12	36.0823	40.6214	12.5800	38.0994	5.5903
			Prediction value	Relative error %	Prediction value	Relative error %
2019	13	45.0492	50.3667	11.8040	47.7188	5.9260
2020	14	59.0303	58.3637	1.1293	59.1627	0.2243
2021	15	76.6671	61.5334	19.7400	77.1866	0.6775
Average simulation relative error			-	10.88	-	15.89
Average prediction relative error			-	10.89	-	2.28
Average total relative error			-	10.88	-	12.75

We build a model using the improved nonlinear optimized GM(1,N) model given by referencing document [38] and then get the following time response equation through calculations:

$$\begin{array}{c} {\hat{x}}^{\left(1\right)}\left(k\right)=1.271086x^{\left(1\right)}\left(k-1\right)+0.00002647118y^{\left(1\right)}\left(k\right)-1.533774. \end{array}$$ (60)

where *x*^(1)^(*t*) is the once accumulated generating column of original sequence and *y*^(1)^(*t*) is the once accumulated generating column of GDP per capita.

We calculate and get the simulation values and prediction values of original sequence in these periods with ${\hat{x}}^{\left(0\right)}\left(t\right)={\hat{x}}^{\left(1\right)}\left(t\right)-{\hat{x}}^{\left(1\right)}\left(t-1\right)$ and the results are shown in Table 3. See Table 3 for the relative errors and average relative errors in the periods.

Next, we build non-grey models. First, we build an ARIMA model. We get the ARIMA(1,2,2) model through calculations:

$$\begin{array}{c} \left(1-B\right)\nabla^{2}{\hat{x}}^{\left(0\right)}\left(k\right)=0.742394+\left(1+0.373654B-0.0294134B^{2}\right)\varepsilon \left(k\right). \end{array}$$ (61)

We calculate and get the simulation and prediction values of original sequence in these periods with the model, and the results are shown in Table 4. See Table 4 for the relative errors and average relative errors in the periods.

**Table 4:** Calculation results of non-grey models for China’s express delivery volume per capita.

Year	No.	*x*^(0)^(*t*)	ARIMA model		Lagging regression model	
			Simulation value	Relative error %	Simulation value	Relative error %
2007	1	0.9096	0.9096	0.0000	-	-
2008	2	1.1395	1.1395	0.0000	-	-
2009	3	1.3922	0.6726	51.6890	2.1450	54.0780
2010	4	1.7443	0.8324	52.2760	2.4348	39.5900
2011	5	2.7225	2.2867	16.0070	2.8867	6.0315
2012	6	4.1829	3.7866	9.4736	4.3906	4.9659
2013	7	6.7191	6.9173	2.9494	6.3144	6.0232
2014	8	10.1414	10.3709	2.2624	9.8247	3.1234
2015	9	14.9403	15.6544	4.7797	14.1598	5.2240
2016	10	22.4684	24.7152	9.9998	20.3340	9.4995
2017	11	28.6091	27.2980	4.5829	30.4238	6.3429
2018	12	36.0823	36.1163	0.0943	36.0887	0.0176
			Prediction value	Relative error %	Prediction value	Relative error %
2019	13	45.0492	44.2466	1.7815	45.1274	0.1737
2020	14	59.0303	53.1543	9.9543	55.8981	5.3061
2021	15	76.6671	62.8044	18.0820	74.5985	2.6982
Average simulation relative error			-	12.84	-	13.49
Average prediction relative error			-	9.94	-	2.73
Average total relative error			-	12.26	-	11.01

Then, we build a lagged regression model. We calculate and get

$$\begin{array}{c} {\hat{x}}^{\left(0\right)}\left(k\right)=1.792897x^{\left(0\right)}\left(k-1\right)-0.7099944x^{\left(0\right)}\left(k-2\right)+0.7478515+\varepsilon \left(k\right). \end{array}$$ (62)

We calculate and get the simulation and prediction values of original sequence in these periods with the model, and the results are shown in Table 4. See Table 4 for the relative errors and average relative errors in the periods.

**Fig 1:**
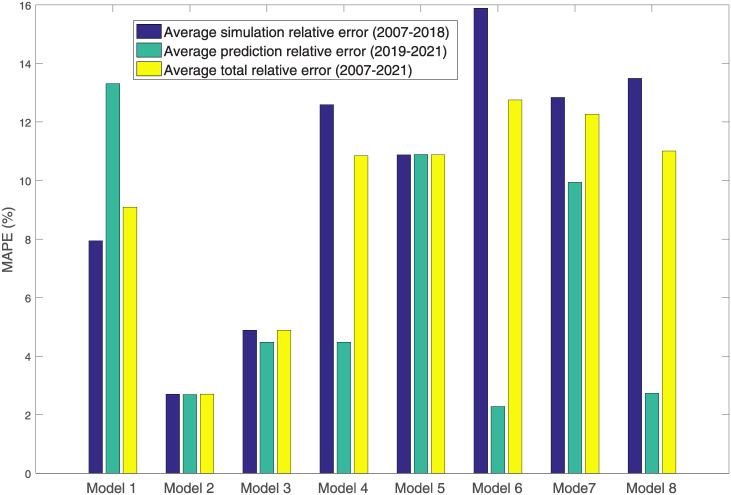
Histogram of relative errors of eight models built for China’s express delivery volume per capita.

Fig 1 is the histogram of relative errors of eight models built for China’s express delivery volume per capita. Models 1~8 are the traditional nonlinear grey Bernoulli model, the model proposed, the model of document [23], the model of document [36], the model of document [37], the model of document [38], the ARIMA model, and the time-lagged regression model. We can see that the model proposed has the highest modeling precision and the precision superior to that of other models.

## Quality evaluations on models

### The robustness evaluation

The robustness evaluation refers to a statistical evaluation on the stability of the model built. We suppose that, for the model, the parameter estimate is *η*, the residua sum of squares is *s*, the sub-sample variance is $m_{s}^{2}$, the degree of freedom is *n*_*s*_ and the population variance is $\delta_{s}^{2}$. We give *η* a small perturbation Δ and record *Q* = *η* + Δ. We consider *Q* as the parameter value of model to get the fitting residual of *r*, and consider corresponding sub-sample variance as $m_{r}^{2}$, freedom degree as *n*_*r*_ and population variance as $\delta_{r}^{2}$ to construct the statistics

$$\begin{array}{c} F=\frac{\delta_{r}^{2}}{\delta_{s}^{2}}\cdot \frac{m_{s}^{2}}{m_{r}^{2}}, \end{array}$$ (63)

*F* ∼ *F*(*n*_*s*_ − 1, *n*_*r*_ − 1). We suppose $H_{0}:\delta_{r}^{2}=\delta_{s}^{2};H_{1}:\delta_{r}^{2}\neq \delta_{s}^{2}$. If it is on the confidence level of *α*,

$$\begin{array}{c} \frac{m_{s}^{2}}{m_{r}^{2}}\leq F_{\frac{\alpha }{2}}\left(n_{s}-1,n_{r}-1\right), \end{array}$$ (64)

*H*_0_ is accepted while *H*_1_ is rejected, i.e. the model is considered as a robust model on the confidence level of 1 − *α*.

For the extended nonlinear grey Bernoulli model established in this paper, we give *η* a small perturbation Δ = 1%, and get $\frac{m_{s}^{2}}{m_{r}^{2}}=\;\;\text{1.0120}$. Because $F_{\frac{\alpha }{2}}\left(n_{s}-1,n_{r}-1\right)=F_{0.01}\left(11,11\right)=4.50$, $\frac{m_{s}^{2}}{m_{r}^{2}}\leq F_{\frac{\alpha }{2}}{\left(n_{s}-1,n_{r}-1\right)}_{\;}$. Therefore, the model is robust.

### The precision evaluation

The precision evaluation is an evaluation on the fitting accuracy of model. It generally analyzes the practical fitting results and calculates some statistics, such as the model’s coefficient of determination $R^{2}=1-\frac{\sum {\left(Y_{t}-{\hat{Y}}_{t}\right)}^{2}}{\sum {\left(Y_{t}-\bar{Y}\right)}^{2}}$, mean absolute relative error $MAPE=\frac{1}{n}\sum_{t=1}^{n}|\frac{Y_{t}-{\hat{Y}}_{t}}{Y_{t}}|$, Theil index of inequality $\mu ={\frac{\sqrt{\frac{1}{n}\sum {\left(Y-\hat{Y}\right)}^{2}}}{\sqrt{\frac{1}{n}\sum Y^{2}+\sqrt{\frac{1}{n}\sum {\hat{Y}}^{2}}}}}_{\;}$ and so on.

For the extended nonlinear grey Bernoulli model established in this paper, we calculate and get the coefficient of determination *R*^2^ = 0.9972 close to 1, the mean absolute relative error *MARE* = 2.70% less than 3%, and the Theil index of inequality *μ* = 0.0272 close to 0, so the model has high precision.

## Results and discussions

Table 1 gives the calculation results of the traditional nonlinear grey Bernoulli model built with the conventional method and the extended nonlinear grey Bernoulli model built with the method proposed. The results show that the nonlinear grey Bernoulli model built with conventional method has big average simulation and prediction relative errors (7.94% and 13.31%, respectively); the extended nonlinear grey Bernoulli model NGBM(1,1,t⌃2,*α*) built with the method proposed has small average simulation and prediction relative errors (2.70% and 2.69%, respectively). It can be seen that the extended nonlinear grey Bernoulli model NGBM(1,1,t⌃2,*α*) built with the method proposed has high precision and is superior to the traditional nonlinear grey Bernoulli model built with the conventional method significantly.

Table 2 gives the calculation results of the grey models built with the methods given by referencing documents [23, 36]. The results show that *x*^(0)^ calculated with the method of referencing document [23] has small average simulation and prediction relative errors (4.89% and 4.88%, respectively) comparatively, but its precision is lower than the calculation precision of the extended nonlinear grey Bernoulli model NGBM(1,1,t⌃2,*α*) built with the method proposed; *x*^(0)^ calculated with the method of referencing document [36] has big average simulation and prediction relative errors (12.59% and 4.48%, respectively), but its precision is lower than that of the nonlinear grey Bernoulli model NGBM(1,1,t⌃2,*α*) built with the method proposed significantly.

Table 3 gives the calculation results of the grey models built with the modeling methods of referencing documents [37, 38]. The results show that *x*^(0)^ calculated with the method of document [37] has big average simulation and prediction relative errors (10.88% and 10.89%, respectively), and its precision is significantly lower than that of the extended nonlinear grey Bernoulli model NGBM(1,1,t⌃2,*α*) proposed; *x*^(0)^ calculated with the method of document [38] has a big average simulation relative error (15.99%), and although with a small average prediction relative error (2.28%), its overall precision is significantly lower than that of the extended nonlinear grey Bernoulli model NGBM(1,1,t⌃2,*α*) built with the method proposed.

Table 4 gives the calculation results of the non-grey models. The results show that the ARIMA model built has big average simulation and prediction relative errors (12.84% and 9.94%, respectively), and its precision is significantly lower than the calculation precision of the extended nonlinear grey Bernoulli model NGBM(1,1,t⌃2,*α*) built with the method proposed; the time-lagged regression model built has a big average simulation relative error (13.49%), and although with a small average prediction relative error (2.73%), the model’s overall precision is significantly lower than that of the extended nonlinear grey Bernoulli model NGBM(1,1,t⌃2,*α*) built with the method proposed.

Therefore, Tables 1 ~ 4 show that the extended nonlinear grey Bernoulli model NGBM(1,1,t⌃2,*α*) built with the method proposed has the precision significantly higher than that of the nonlinear grey Bernoulli model built with the traditional method and superior to that of the models built with the methods of referencing documents [23, 36–38], the ARIMA model and the time-lagged regression model. It indicates that the extended model and method proposed in the paper have high reliability and effectiveness.

The express delivery volume per capita is a high-growth sequence, and studies have shown that general grey models are adapted to the variations of low-growth sequence. The common grey modeling methods rarely transform the accumulated generating sequence. Some scholars have extended the grey action of grey model, but the extended model failed to present the variation law of high-growth sequence. The paper improves the accumulated generating sequence to make the high-growth sequence adapt to the variation law of nonlinear grey Bernoulli model, and extends the grey action of nonlinear grey Bernoulli model simultaneously to make the improved model present the variation characteristics of high-growth sequence. In addition, we use the cubic spline interpolation function as the background value to make the model have better fitting elasticity. We optimize the parameters in the three aspects simultaneously, to make the model have higher precision overall.

## Conclusion

(1) To increase the nonlinear grey Bernoulli model’s prediction precision and increase the data’s adaptability to the model, the paper transforms the accumulated generating sequence of original time sequence, *x*^(0)^ = {*x*^(0)^(1), *x*^(0)^(2), ⋯, *x*^(0)^(*n*)}, i.e. the new accumulated generating sequence is *x*^(*r*)^ = {*x*^(*r*)^(1), *x*^(*r*)^(2), ⋯, *x*^(*r*)^(*n*)} in which $x^{\left(r\right)}\left(k\right)=\sum_{i=1}^{k}\frac{x^{\left(0\right)}\left(i\right)}{g[\text{sin}\left(\frac{\pi }{2}\left(1+he^{-vi}\right)\right]}$. Because the original accumulated generating sequence is an exception of new accumulated generating sequence, for a specific grey model, if it meets the rules of traditional accumulated generating sequence, it must meet the rules of new accumulated generating sequence. Therefore, improving the accumulated generating sequence of *x*^(0)^(*t*) can improve modeling precision.

(2) To improve the model’s prediction precision, the paper extends the structure of traditional nonlinear grey Bernoulli model by extending grey action (*x*^(*r*)^(*t*))^*α*^ to be (*b*_0_+ *b*_1_*t* + *b*_2_*t*^2^+ ⋯ + *b*_*p*_*t*^*p*^)(*x*^(*r*)^(*t*))^*α*^ to make the model have the generality to be adaptive to more variations of data. The extended nonlinear grey Bernoulli model is recorded as EGPM(1,1,t⌃p,*α*).

(3) To improve the model’s prediction precision, the paper uses the interpolation function as the background value function and chooses the cubic spline interpolation function as the interpolation function to have more flexible background values. The four background values are *z*^(*i*)^(*k*) = *a*_*ik*_[1 − *w*_*i*_]^3^ + *b*_*ik*_[1 − *w*_*i*_]^2^ + *c*_*ik*_[1 − *w*_*i*_] + *d*_*ik*_, (0 ≤ *w*_*i*_ ≤ 1), and (*a*_*ik*_, *b*_*ik*_, *c*_*ik*_, *d*_*ik*_), (*i* = 1, 2, 3, 4;*k* = 2, 3⋯, *n*) is the cubic spline interpolation function coefficient.

(4) The paper gives the parameter optimization method and time response equation of extended nonlinear grey Bernoulli model NGBM(1,1,t⌃2,*α*). The paper improves the model in three aspects and the parameters in the three aspects are optimized simultaneously, improving the model’s simulation and prediction precision greatly.

(5) Using the modeling method of extended nonlinear grey Bernoulli model NGBM(1,1,t⌃p,*α*) proposed, the paper builds a grey model for China’s express delivery volume per capita. Results show that the model build has the simulation and prediction precision higher than that of traditional nonlinear grey Bernoulli model significantly. The extended nonlinear grey Bernoulli model NGBM(1,1,t⌃2,*α*) has an average simulation relative error of 2.70% and an average prediction relative error of 2.69% which are both small. The grey modeling for rapid growth sequence like China’s express delivery volume per capita has such high precision, indicating the method proposed is highly scientific.

(6) To compare the model proposed with the nonlinear grey Bernoulli models proposed by other referencing documents in terms of modeling precision, the paper makes modeling calculations for the methods proposed by referencing documents [23, 36–38]. Calculation results show that the model built with the modeling method proposed has the precision significantly superior to that of the models built with the methods of the documents. The comparison results of modeling results with two non-grey models also show the model proposed has the highest precision. Therefore, the model built with the method proposed has high reliability. The idea and method offered in the paper have important significance for the in-depth study and wide applications of nonlinear grey Bernoulli model.
